# A High-Capacity Steganography Algorithm Based on Adaptive Frequency Channel Attention Networks

**DOI:** 10.3390/s22207844

**Published:** 2022-10-15

**Authors:** Shanqing Zhang, Hui Li, Li Li, Jianfeng Lu, Ziqian Zuo

**Affiliations:** 1 School of Computer Science and Technology, Hangzhou Dianzi University, Hangzhou 310018, China; 2 Key Laboratory of Brain Machine Collaborative Intelligence of Zhejiang Province, Hangzhou 310018, China

**Keywords:** image steganography, generative adversarial networks, discrete cosine transform, attention mechanism

## Abstract

Deep learning has become an essential technique in image steganography. Most of the current deep-learning-based steganographic methods process digital images in the spatial domain. There are problems such as limited embedding capacity and unsatisfactory visual quality. To improve capacity-distortion performance, we develop a steganographic method from the frequency-domain perspective. We propose a module called the adaptive frequency-domain channel attention network (AFcaNet), which makes full use of the frequency features in each channel by a fine-grained manner of assigning weights. We apply this module to the state-of-the-art SteganoGAN, forming an Adaptive Frequency High-capacity Steganography Generative Adversarial Network (AFHS-GAN). The proposed neural network enhances the ability of high-dimensional feature extraction through overlaying densely connected convolutional blocks. In addition to this, a low-frequency loss function is introduced as an evaluation metric to guide the training of the network and thus reduces the modification of low-frequency regions of the image. Experimental results on the Div2K dataset show that our method has a better generalization capability compared to the SteganoGAN, with substantial improvement in both embedding capacity and stego-image quality. Furthermore, the embedding distribution of our method in the DCT domain is more similar to that of the traditional method, which is consistent with the prior knowledge of image steganography.

## 1. Introduction

To secure information, there is an urgent need to solve the problems of privacy protection and information security in the process of multimedia communication. Image steganography is the process of hiding the message into the cover image in a way that is invisible to the human eyes. It is divided into the traditional image steganography method and the deep learning-based method.

The traditional steganography method hides the message into the spatial or frequency domain of the cover image by a specific embedding method and obtains good transparency and security. However, the amount of hidden data is small and cannot meet the requirements of high-volume image steganography tasks, such as Pevn’y [1] who could only achieve a payload of about 0.4 bits per pixel. The statistical feature changes caused by traditional methods can be easily detected by automatic steganalysis tools and, in extreme cases, by the human eyes.

With the wave of deep learning hitting in the last decade, a new class of image steganography methods emerged. Hayes & Danezis [2], Baluja [3], Zhu [4], and others proposed some new image steganography methods that break the limitations of traditional methods in terms of hiding capacity and the performance of these methods show the superior competitiveness than traditional methods. SteganoGAN [5] proposed by Zhang [5] adds a critic to the encoder-decoder-based steganographic framework and designs the loss function from multiple perspectives, using three variants of the encoder to cope with different loading scenarios: basic, residual and dense. SteganoGAN substantially improved the hidden capacity while ensuring visual quality, achieving up to 4.4 bpp on the COCO dataset. However, it only attains 2.63 bpp on the Div2k dataset, suggesting an insufficient generalization capability. The research on image steganography based on deep learning is becoming more and more popular, and it is very important to continue to study and improve the deep learning network model in order to achieve sufficiently high embedding capacity.

The study of traditional image steganography methods have been focused in the frequency domain for a long time. There are many methods proposed to embed information in frequency domains, such as discrete Fourier transform (DFT) domain [6], discrete cosine transform (DCT) domain [7], and discrete wavelet transform (DWT) domain [8]. In the literature [7,9,10] and other traditional image steganography methods based on the DCT domain, information is usually chosen to be embedded in the low and middle-frequency bands of the DCT domain to balance transparency and accuracy.

**Figure 1:**
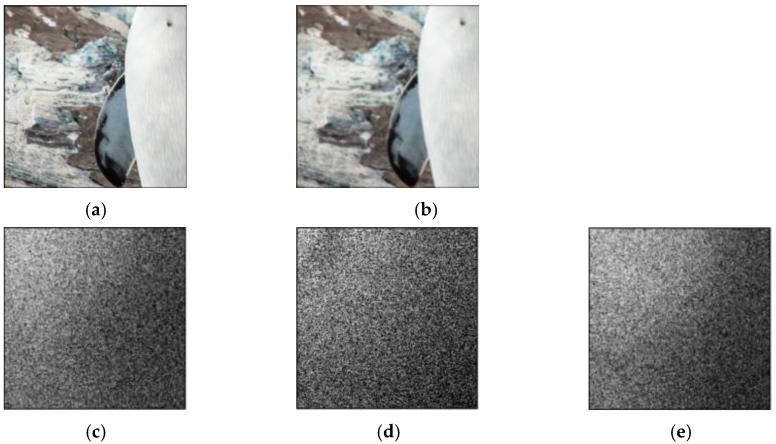
Residual distribution of SteganoGAN in the DCT domain. (**a**) Cover Image; (**b**) Steganographic Image; (**c**) R-channel DCT Domain Residual; (**d**) G-channel DCT Domain Residual; (**e**) B-channel DCT Domain Residual.

Deep learning-based steganographic networks for digital images need to balance the complexity and performance of the model in order to pursue the usability and practicality of the network model. Usually, few parameters and simple network structures as possible are used to speed up the convergence of the model. The residuals of the stego image and cover image in the DCT domain for the SteganoGAN method are shown in Figure 1. The messages are embedded centrally in the low-frequency band, and the feature information in the middle and high-frequency bands is not fully utilized. This leads to the problem of insufficient generalization ability on the Div2K dataset.

**Figure 2:**
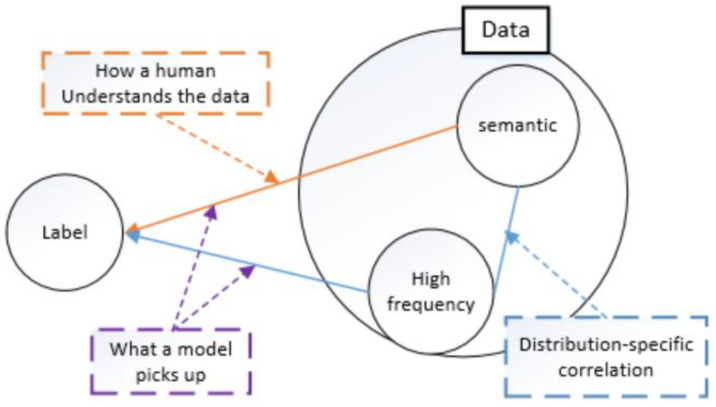
The difference between CNN and human understanding of the data.

Wang et al. [11] investigated the relationship between the spectrum of image data and the generalization behavior of CNNs, where human understanding of data is often based on the semantic information represented in the low-frequency segment of the data, and CNNs can capture the high-frequency components of images, as shown in Figure 2. However, CNNs are not able to selectively utilize the high-frequency components, which not only contain information related to the distribution characteristics of the data, but also may contain noisy components. Therefore, how to make the neural network make better use of high-frequency components remains to be studied.

Feature selection plays a crucial role in the performance of CNNs, where one convolutional kernel corresponds to one channel, and features (feature maps or channels) can be selected dynamically by adjusting the weights associated with the convolutional kernels. The terms “feature map” and “channel” will be used interchangeably in this paper, as many researchers use the term “channel” to represent a feature map. Some feature maps play little or no role in the target task [12], so a large set of features may produce noise effects and lead to a degradation in the performance of the network; as such, it is still important to pay attention to how to reasonably enhance the feature map information when building a CNNs structure. Attention in CNNs uses only a small number of parameters to perform feature enhancement for deep learning networks, and the goal is to selectively focus on some important information for feature extraction, corresponding to different feature dimensions with spatial attention, channel attention, and hybrid attention.

Channel attention methods are used for feature selection and enhancement. Since the construction of the channel importance weight function is limited by the computational overhead, a preprocessing process is required to first compress each feature map into a scalar, which is then used to compute the weights for that channel. The global average pooling (GAP) used by SENet [12] is popular among deep learning developers due to its simplicity and efficiency. However, it suffers from lack of feature diversity when processing different inputs, wasting rich input feature information. FcaNet [13] extends frequency-domain features to preprocessing, using multiple but limited frequency components in preprocessing, integrating multiple frequency components into the channel attention network. However, there is the problem of underutilization of single-channel information, where each single channel can only pay attention to one predefined frequency component, although globally multiple frequency component information is utilized. However, locally, a single channel can still only utilize one frequency component, which wastes a lot of useful feature information.

We propose an Adaptive Frequency-Domain Channel Attention Network (AFcaNet) by assigning weights to the frequency-domain coefficients of the feature maps at a finer granularity, and then making full use of the frequency-domain features in a single channel. Applying this to SteganoGAN, we propose the Adaptive Frequency High-capacity Steganography Generative Adversarial Network (AFHS-GAN) by superimposing densely connected convolutional blocks and adding low-frequency loss functions. The experimental results show that our method exhibits better generalization on Div2K dataset compared to SteganoGAN, with substantial improvement in both hidden capacity and image quality. Through experimental analysis, the embedding distribution of our method in the frequency domain is more like that of the traditional method, which is consistent with the a priori knowledge of image steganography.

The main innovations of this paper are as follows:

- We propose the adaptive frequency-domain channel attention network AFcaNet, replacing the fixed frequency-domain coefficients in FcaNet with weighted frequency-domain coefficients for enhancing the feature extraction of individual channels.
- We apply AFcaNet in image steganography networks and optimize the number and location of AFcaNet additions to ensure model reliability while enhancing important frequency-domain features.
- A low-frequency discrete cosine loss function is proposed. To guide the adaptive frequency-domain channel attention, the frequency-domain loss is added to reduce the modification of the low-frequency region of the image, which in turn improves the image quality.
- The deep network built based on densely connected convolutional blocks for stacking is more powerful for high-dimensional image feature extraction.

The remaining structure of this paper is mainly as follows: our methodology is presented in Section 2, the experimental results are analyzed in Section 3, and the conclusions are presented in Section 4.

## 2. Our Method

**Figure 3:**
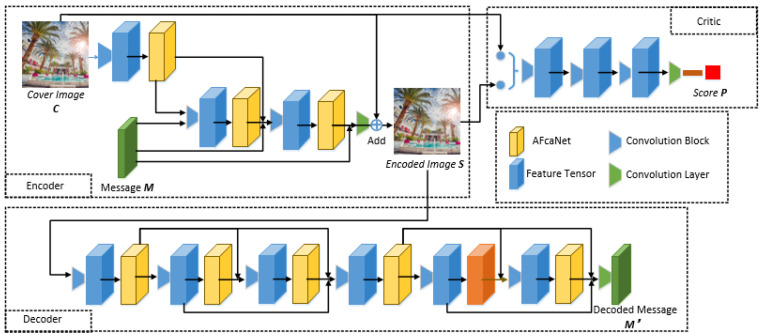
The network structure of AFHS-GAN.

The image steganographic adversarial network AFHS-GAN is based on adaptive frequency-domain attention. This model consists of four main modules: Adaptive Frequency Channel Attention Network (AFcaNet): adaptively extracting the frequency-domain features of each feature map, calibrating the channel weights of the feature tensor, enhancing the important feature maps; Encoder: using convolution layer, dense connection and AFcaNet, fusing cover image C and message M, outputting steganographic image S; Decoder: using convolution layer, dense connection and AFcaNet to recover the message $M^{\prime }$ in steganographic image; Critics: simulating steganographic analysis, discriminating the naturalness of the input image and outputs the score P, which improves the security of the steganographic image generated by the encoder by confronting with the encoder. The flowchart of the method in this section is shown in Figure 3.

### 2.1. AFcaNet

**Figure 4:**
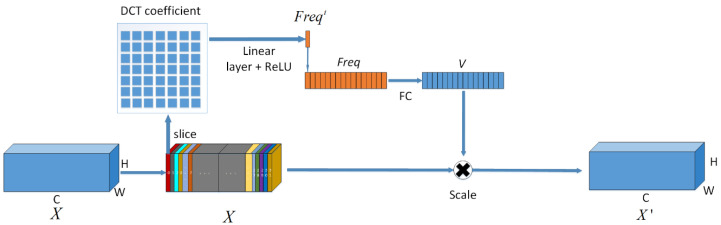
The network structure of AFcaNet.

Adaptive Frequency-domain Channel Attention AFcaNet is a channel attention method for use in CNNs, the network structure is shown in Figure 4. The input to the module is the feature tensor$\;X\;\in \;{\mathbb{R}}^{\;\;C\times H\times W}$, Generate a weight vector by a series of feature extraction $V\;\in \;{\mathbb{R}}^{\;\;C}$. Using vector V to calibrate and augment the tensor X along the channel-wise, the output feature tensor is $X^{\prime }\in \;{\mathbb{R}}^{\;\;C\times H\times W}$.

Step 1: There are C slices along channel-wise of the tensor X $\left[X^{0},\;X^{1},\ldots ,X^{C-1}\right]$. Each slice$\;X^{i}\;\in \;{\mathbb{R}}^{\;\;H\times W},\;i\;\in \;\left\{0,1,\ldots ,C-1\right\}$ is mapped to the 2D-DCT domain. A complete mapping to a 2D-DCT domain of the same size H×W would consume a lot of computational resources. Therefore, the 2D-DCT domain is compressed, and each dimension is divided into 7 equal parts, and 7×7 parts are obtained by dividing. Only the lowest frequency component is taken for each W/7×H/7 part. To map to the compressed 2D-DCT domain, only 49 DCT components need to be computed for each slice and concatenated into a 49-dimensional vector $DCT^{7\times 7}\;\in \;{\mathbb{R}}^{\;\;49}$, can be written as:

(1) $$B_{h,w}^{i,j}=\cos(\frac{\pi h}{H}\left(i+\frac{1}{2}\right))\cos(\frac{\pi w}{W}\left(j+\frac{1}{2}\right))$$

(2) $$\begin{array}{l} DCT^{i}=2DDCT^{u,v}\left(X^{i}\right),\;u=i/7,\;v=i\mathrm{mod}7\\ =\overset{H-1}{\underset{h=0}{\sum }}\overset{W-1}{\underset{w=0}{\sum }}X_{:,h,w}^{i}B_{h,w}^{u,v}\;s.t.i\;\in \;\left\{0,1,\cdots ,n-1\right\} \end{array}$$

(3) $$DCT^{7\times 7}=Cat\left(\left[DCT^{0}\left(X^{i}\right),DCT^{0}\left(X^{i}\right),\cdots ,DCT^{48}\left(X^{i}\right)\right]\right)$$

Step 2: The i-th tensor slice $X^{i}$ is mapped to the compressed frequency domain, and the feature scalar $Freq^{i}$ is output as the preprocessing result of the slice by the full connection and ReLU activation function, can be written as:

(4) $$Freq^{i}=ReLU\left(fc_{49\to 1}\left(DCT^{7\times 7}\right)\right)$$

(5) $$Freq=Cat\left(\left[Freq^{0},Freq^{1},\cdots ,Freq^{n-1}\right]\right)$$

where $fc_{49\to 1}\;$ is used to denote a fully connected layer with input size 49 and output size 1, and ReLU denotes the ReLU activation function.

Step 3: Let sigmoid denote the sigmoid activation function, fc denote the fully connected layer and generate the weight vector $Weight\;\in \;{\mathbb{R}}^{C}$.

(6) Weight=sigmoid(fc(Freq))

Step 4: Weight vector sliced by channel dimension $Weight=\left[Weight_{1},Weight_{2},\cdots ,Weight_{C}\right]$ and feature tensor $X=\left[X_{1},X_{2},\cdots ,X_{C}\right]$, Each slice in the enhanced tensor $X^{\prime }=\left[{X_{1}}^{\prime },{X_{2}}^{\prime },\cdots ,{X_{C}}^{\prime }\right]$ can be written as:

(7) $${X_{C}}^{\prime }=F_{scale}\left(X_{C},Weight_{C}\right)=X_{C}\;Weight_{C}$$

To facilitate the following representation of the use of AFcaNet, the function af_att (Adaptive Frequency Channel Attention) is used to represent the above four steps, and the feature tensor *X*^′^ after channel calibration can be written as:

(8) $$X^{\prime }=af\_att\left(X\right)$$

In our experiments, the input feature tensor X is of size 32×360×360 where the number of channels C=32, height H=360 and width W=360.

### 2.2. Encoder

The network structure of the encoder is based on the dense encoder improvement in the SteganoGAN network. The AFcaNet is inserted in the encoder and the attention network can be enhanced with features having a small number of parameters. The encoder network consists of three convolutional blocks and three AFcaNet and one convolutional layer, where the specific structures of the three convolutional blocks are: convolutional layer: 32 convolutional kernels with size 3×3, 1 step, and 1 padding; LeakyReLU; Batch normalization. The AFcaNet is inserted to enhance the features after each convolutional block, and the feature tensor of the last layer of the network is tensor-added with the cover image to generate a steganographic image.

The inputs to the encoder network are the cover image C and the message $M\;\in {\left\{0,1\right\}}^{D\times W\times H}$, D denotes the number of bits embedded in each pixel in the cover image. First extract the features of cover image C to obtain the tensor a. Then use AFcaNet to recalibrate the channel-wise feature of tensor a to get $a^{\prime }$, and let $af\_att_{i},i\;\in \;\left\{1,2,3\right\}$ denote the i-th AFcaNet in the network, can be written as:

(9) $$a=Conv_{3\to 32}\left(C\right)$$

(10) $$a^{\prime }=af\_att_{1}\left(a\right)$$

where $Conv_{3\to 32}$ denotes a convolutional layer or convolutional block that maps an input tensor C of depth 3 to a tensor feature *a* of the same width and height but depth 32.

The message M is concatenated with the tensor $a^{\prime }$ in the channel-wise, and the tensor *b* is obtained after the convolution block. AFcaNet recalibrates the channel features of b to obtain 

:

(11) $$b=Conv_{32+D\to 32}\left(Cat\left(a,M\right)\right)$$

(12) $$b^{\prime }=af\_att_{2}\left(b\right)$$

The network adds additional dense connections between the convolutional blocks, connecting the previously generated feature maps to the convolutional blocks afterwards, and concatenating them with the feature tensor afterwards to form a new tensor. In Figure 3, the trapezoid indicates the convolutional block, and two or more arrows merge to indicate the concatenation operation. This connection is inspired by the DenseNet architecture of Huang et al. [14], which has been shown to feature reuse and mitigate the problem of gradient disappearance. Thus, dense connections are utilized to improve embedding efficiency. It can be written as:

(13) $$c=Conv_{64+D\to 32}\left(Cat\left(a,b,M\right)\right)$$

(14) $$c^{\prime }=af\_att_{3}\left(c\right)$$

(15) $$d=Conv_{96+D\to 3}\left(Cat\left(a,b,c,M\right)\right)$$

(16) E(C,M)=C+d

Finally, the steganographic image S=E(C,M) is output with the same resolution and number of channels as the cover image C.

### 2.3. Decoder

Decoder improves the representational capability of the network by increasing the depth of the network, which uses a double dense connection structure for more deep feature extraction capability. Input is the steganographic image S generated by the encoder. Output is the predicted message $M^{\prime }$. The decoder contains 1 convolutional layer, 6 convolutional blocks and 6 AFcaNet, the specific composition of the convolutional blocks is the same as that of the encoder. can be written as:

First, the initial feature extraction of the steganographic image S:

(17) $$a=Conv_{3\to 32}\left(S\right)$$

(18) $$a^{\prime }=af\_att_{4}\left(a\right)$$

Second, the first densely-connected layer:

(19) $$b=Conv_{32\to 32}\left(a\right)$$

(20) $$b^{\prime }=af\_att_{5}\left(b\right)$$

(21) $$c=Conv_{64\to 32}\left(Cat\left(a^{\prime },b^{\prime }\right)\right)$$

(22) $$c^{\prime }=af\_att_{6}\left(c\right)$$

(23) $$d=Conv_{96\to 32}\left(Cat\left(a^{\prime },b^{\prime },c^{\prime }\right)\right)$$

(24) $$d^{\prime }=af\_att_{7}\left(d\right)$$

Finally, the second densely-connected layer:

(25) $$b_{2}=Conv_{32\to 32}\left(d^{\prime }\right)$$

(26) $${b_{2}}^{\prime }=af\_att_{8}\left(b_{2}\right)$$

(27) $$c_{2}=Conv_{64\to 32}\left(Cat\left(d^{\prime },{b_{2}}^{\prime }\right)\right)$$

(28) $${c_{2}}^{\prime }=af\_att_{9}\left(c_{2}\right)$$

(29) $$D\left(S\right)=Conv_{96\to D}\left(Cat\left(d^{\prime },{b_{2}}^{\prime },{c_{2}}^{\prime }\right)\right)$$

The predicted message $M^{\prime }=D\left(S\right)$ generated by the decoder should be as similar as possible to the input message M  of the encoder.

### 2.4. Critics

Based on the idea of GAN, a critic that works against the encoder is used to guide the encoder to generate a more realistic image. The network structure of the critic is the same as SteganoGAN, consisting of 3 convolutional blocks and 1 convolutional layer. The last convolutional layer output is followed by using adaptive mean pooling. The input is cover image C or steganographic image S and the output is a scalar score P. It can be written as:

(30) $$a=Conv_{32\to 32}\left(Conv_{32\to 32}\left(Conv_{3\to 32}\left(S\right)\right)\right)$$

(31) $$C\left(S\right)=Mean\left(Conv_{32\to 1}\left(a\right)\right)$$

The symbol Mean denotes the adaptive mean spatial pooling operation, which calculates the mean value of each feature map.

### 2.5. Training

In SteganoGAN, the encoder-decoder network and the critic network are iterated and optimized by joint training, and each of the three modules has a loss function corresponding to the different target tasks. To balance the different task objectives, the joint three loss functions are used to guide the optimization direction during the training iterations of the encoder-decoder network. Let the italicized capital letters *C*, *S*, and *M* denote cover image, steganographic image, and message. Characters E, D, C denote encoder, decoder, and critic. The basic loss functions are loss function *ℒ_d_* for decoder, loss function ${ℒ}_{e}$ for encoder, and loss function ${ℒ}_{c}$ for critic.

{(32)ℒd=EC~ℙcCrossEntropy(D(E(C,M)),M)(33)ℒe=EC~ℙc13×W×H||C−E(C,M))||22(34)ℒc=EC~ℙcC(E(C,M))

In addition to using the loss function in SteganoGAN described above, we propose a low-frequency loss ${ℒ}_{freq}$ to enhance the performance of the network. Inspired by the literature [15], which verified in the wavelet domain that messages hidden in high-frequency components are less detectable than those in low-frequency components. ${ℒ}_{freq}$ reduces the proportion of messages hidden in the low-frequency band, making the DCT low-frequency coefficients of the steganographic image more like the DCT low-frequency coefficients of the cover image and improving the image quality. Setting $DCT^{i}$ to denote the frequency component at position (i/7, imod7), the low-frequency discrete cosine loss ${ℒ}_{freq}$ is defined as follows, where $x^{i}$ is used to denote the i-th channel of x, which in this case refers to the RGB three-channel of the color image.

(35) $${ℒ}_{freq}\left(\theta \right)=\overset{3}{\underset{n}{\sum }}\overset{7}{\underset{i=0}{\sum }}\left|DCT^{i}\left(x_{cover}^{\left(n\right)}\right)-DCT^{i}\left(x_{stego}^{\left(n\right)}\right)\right|$$

The relationship between ${ℒ}_{freq}$ and the basic loss is balanced using the weight factor λ. Therefore, the overall objective of the encoder-decoder network training is to minimize the loss:

(36) $$\mathrm{minimize}\;{ℒ}_{d}+{ℒ}_{e}+{ℒ}_{c}+\lambda \cdot {ℒ}_{freq}$$

Minimizing Wasserstein loss to train critics networks.

(37) $${ℒ}_{r}=E_{C\sim {\mathbb{P}}_{c}}C\left(X\right)-E_{C\sim {\mathbb{P}}_{c}}C\left(E\left(C,M\right)\right)$$

Each iteration matches the cover image C to a data tensor M. The tensor M  consists of a randomly generated sequence (D×W×Hbits) that is sampled from a Bernoulli distribution M~Ber(0.5).

## 3. Experiments

### 3.1. Experimental Setup

In order to validate the superiority of our method in a fair situation, we implemented experiments with our method and the publicly available source code of SteganoGAN on the same machine. This machine has a dual-core CPU, i9-10900X@3.70GHZ, with 64.00 GB of RAM. All experiments are conducted in this machine with Python 3.8.5, Pytorch 1.0.0 and Nvidia GTX 3090 graphics card. Both methods were trained on the Div2K dataset, which consists of 1000 high-quality pixel-level images. Our method uses a random cropping method to crop the input images to 3×360×360 in order to alleviate the overfitting problem in training. the initial learning rate of the Adam optimizer is $1e^{-4}$. The model training convergence takes about 28 h and 600 epochs of iterations.

We evaluate the performance of our method using the same four objective evaluation metrics as SteganoGAN: peak signal-to-noise ratio (PSNR), structural similarity Index (SSIM), Reed-Solomon bits per pixel (RS-BPP), and decoding accuracy (ACCURACY). Eight different data depths D∈{1,2,…8} are used in the experiments to denote the capacity of D bits per pixel. It is important to note that the steganography method has an unavoidable trade-off between payload and image quality, and an increase in payload will inevitably lead to a decrease in image quality.

(1) Accuracy: the percentage of correct bits in the decoded message. Among the total N bits of the decoded message $M^{\prime }$, the correct T bits and the incorrect F bits, T+F=N.

(38) $$Accuracy=\frac{T}{N}$$

(2) RS-BPP: given a piece of binary messages M of length k, the Reed-Solomon error correction code generates a binary error correction message of length n(n ≥ k) which allows $\frac{n-k}{2}$ bit errors and can correct the bit errors [16]. D  is the number of bits per pixel for the attempted embedding and can be written as:

(39) $$RS-BPP=D\times \frac{k}{n}$$

(3) PSNR: the peak signal-to-noise ratio is used to measure the degree of distortion of a steganographic image and has been shown to be like the average opinion scores derived by human experts [17]. Given two images of size (W,H) of X and Y, and a scaling factor sc that denotes the maximum possible difference in each pixel, the PSNR can be calculated from the mean squared error MSE as:

(40) $$MSE=\frac{1}{WH}\overset{W}{\underset{i=1}{\sum }}\overset{H}{\underset{j=1}{\sum }}{\left(X_{i,j}-Y_{i,j}\right)}^{2},$$

(41) $$PSNR=20\cdot \log_{10}(sc)-10\cdot \log_{10}(MSE)$$

(4) SSIM: widely used in the broadcast industry to measure image and video quality [17]. Given two images X and Y, SSIM can be defined by the mean $\mu_{X}$ and $\mu_{Y}$, variance $\sigma_{X}^{2}$ and $\sigma_{Y}^{2}$ and covariance $\sigma_{\mathrm{XY}}^{2}$ of these two images.

(42) $$SSIM=\frac{\left(2\mu_{X}\mu_{Y}+k_{1}R\right)\left(2\sigma_{XY}+k_{2}R\right)}{\left(\mu_{X}^{2}+\mu_{Y}^{2}+k_{1}R\right)\left(\sigma_{X}^{2}+\sigma_{Y}^{2}+k_{2}R\right)}$$

The SSIM is usually calculated by default setting $k_{1}=0.01$ and $k_{2}=0.03$, then obtaining a range of values SSIM ∈ [−1.0,1.0], where 1.0 means the images are identical.

### 3.2. Analysis of Network Experiment Results

To verify the similarity of the embedding position of our method in the DCT domain with the traditional steganography method, we train our method and save the network model after convergence. Then we visualize the weight parameters of the first linear connectivity layer of AFcaNet, representing the degree of enhancement for different frequency-domain coefficients. The input of this layer is 49 DCT frequency components and the output is a feature scalar. The target task of this layer is to learn the weight of each frequency component.

**Figure 5:**
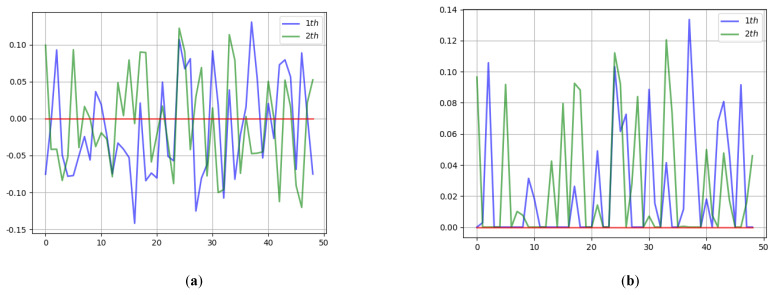
Results of the first two AFcaNet weights of the encoder. (**a**) Linear Layer Weight; (**b**) After ReLU Weight.

Our method uses a total of 9 AFcaNet, with 3 attentions used in the encoder and 6 attentions used in the decoder. The weight learning results in the front two AFcaNet in the encoder are shown in Figure 5. The figure shows that the weights of the frequency components have a wavy distribution. Because the adjacent frequency coefficients carry similar energy in the initial stage of feature extraction and information fusion, the wavy weight distribution helps to remove redundant information.

**Figure 6:**
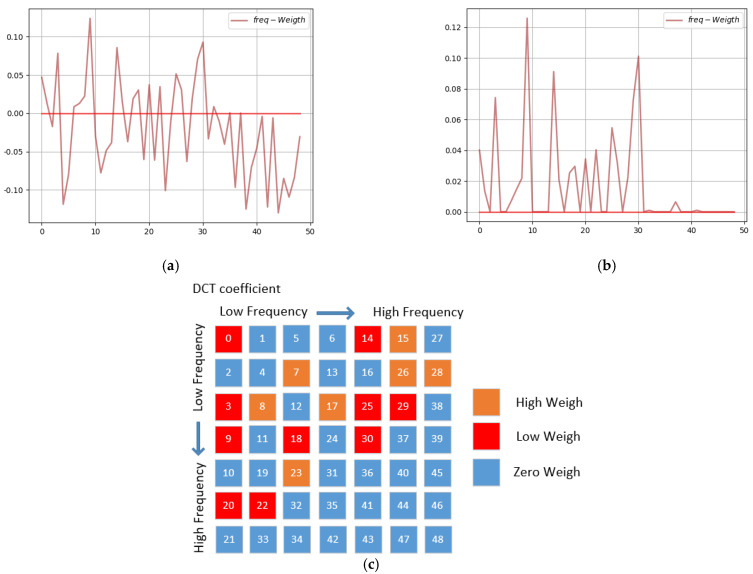
Results of the third AFcaNet weight of the encoder. (**a**) Linear Layer Weight; (**b**) After ReLU Weight; (**c**) The (**b**) mapping in 2D-DCT domain.

The 3rd AFcaNet in the encoder is the last layer of the network, so the weight result of this layer AFcaNet can roughly represent the frequency-domain band in which the message is embedded, as shown in Figure 6. It can be seen from the figure that the embedding position of the 3rd AFcaNet in the DCT domain is concentrated in the middle and low-frequency bands, and the high-frequency band is almost not selected. This is similar to the selection of frequency-domain embedding locations by traditional steganography methods based on the frequency domain, indicating that the learning logic of our method is consistent with the a priori knowledge of image steganography.

**Figure 7:**
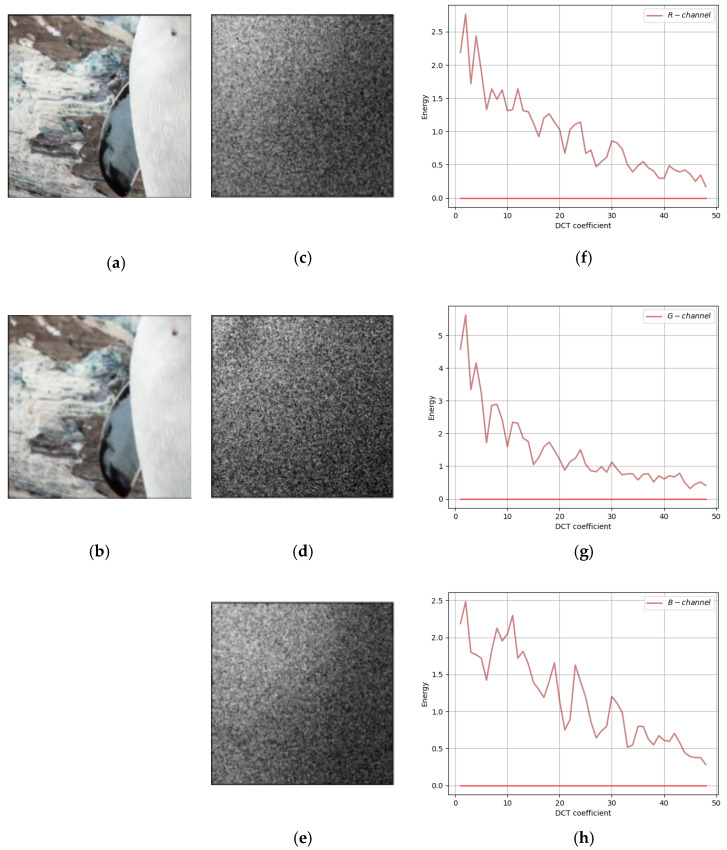
A frequency-domain residual analysis of SteganoGAN. (**a**) Cover Image; (**b**) Steganographic Image; (**c**) R-channel DCT Domain Residual; (**d**) G-channel DCT Domain Residual; (**e**) B-channel DCT Domain Residual; (**f**) R-channel DCT residual curves; (**g**) G-channel DCT residual curves; (**h**) B-channel DCT residual curves.

**Figure 8:**
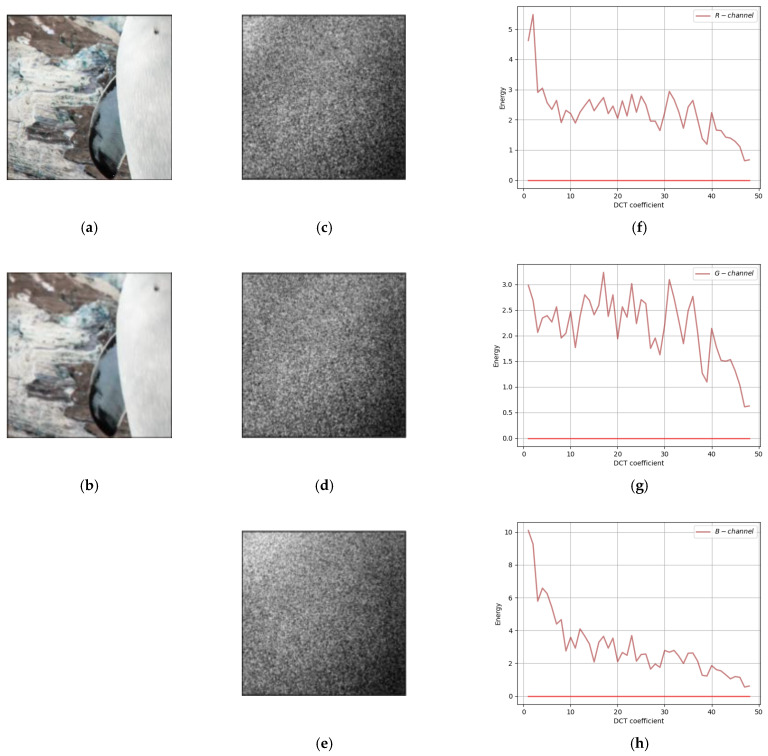
A frequency-domain residual analysis of F5. (**a**) Cover Image; (**b**) Steganographic Image; (**c**) R-channel DCT Domain Residual; (**d**) G-channel DCT Domain Residual; (**e**) B-channel DCT Domain Residual; (**f**) R-channel DCT residual curves; (**g**) G-channel DCT residual curves; (**h**) B-channel DCT residual curves.

**Figure 9:**
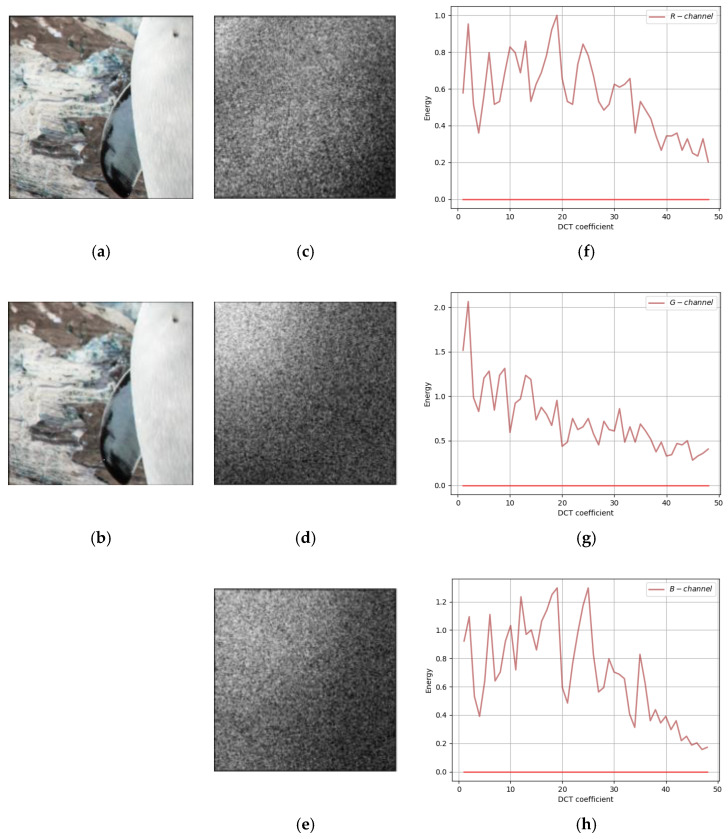
A frequency-domain residual analysis of our method. (**a**) Cover Image; (**b**) Steganographic Image; (**c**) R-channel DCT Domain Residual; (**d**) G-channel DCT Domain Residual; (**e**) B-channel DCT Domain Residual; (**f**) R-channel DCT residual curves; (**g**) G-channel DCT residual curves; (**h**) B-channel DCT residual curves.

Comparing our method with the traditional F5 method and SteganoGAN, the residuals of cover image and steganographic image in DCT Domain are shown in Figure 7, Figure 8 and Figure 9, respectively. In all three figures, the first column shows the input cover image and the output steganographic image. The second column shows the DCT domain residuals of the two images on the three channels of RGB, with the DC component removed and image enhanced for easy observation. The third column is the plot of the second column on the two-dimensional axes, the horizontal coordinate is the 49 frequency components, and the vertical coordinate is the magnitude of the carried energy of the frequency components.

The embedding position of the message can be seen from the residual plot. First, the frequency-domain residual analysis of the experimental results of the SteganoGAN is shown in Figure 7. The figure shows that the residuals are mainly concentrated in the low-frequency band, i.e., the message is mainly embedded in the low-frequency band.

Secondly, the frequency-domain residual analysis of the experimental results of the traditional method F5 is shown in Figure 8. The second column of the figure (Figure 8c–e) has a more dispersed area of white point distribution, except for the concentrated white point in the upper left corner, and the middle area shows a band distribution. The third column of the figure (Figure 8f–h) shows that there are several peak points in the middle frequency band, indicating that the residuals of the F5 method are not only distributed in the low-frequency band, but also have some distribution in the middle frequency band.

Finally, the frequency-domain residual analysis of our method is shown in Figure 9. The residual plot in the second column (Figure 9c–e) has a band-like distribution in the main region of the white dots and in the upper left corner. The frequency-domain energy plot in the third column also shows that the curve peaks exist in the middle frequency band and low-frequency band. Compared with SteganoGAN, our method has a wider distribution in the frequency domain, which is more like the embedding logic of traditional methods. Our method sacrifices some of the robustness of low-frequency embedding, which results in higher quality of the generated steganographic image S and higher accuracy of decoded message $M^{\prime }$.

### 3.3. Performance Comparison

A comparison of the performance of our method with SteganoGAN at different hiding capacities is shown in Table 1. In terms of hiding capacity, SteganoGAN achieves the highest payload at hiding capacity D=4, which can carry 3.42 bits per pixel. And our method achieves the highest payload at the hidden capacity D=8, carrying 5.22 bits per pixel, which improves the highest payload by 1.8 bits per pixel compared to SteganoGAN. In terms of transparency, the PSNR metric of our method at higher payload at hidden capacity D ≤ 4 is on average 2.32 higher than that of SteganoGAN, while at high capacity our method improves the embedding capacity at the expense of transparency.

**Figure 10:**
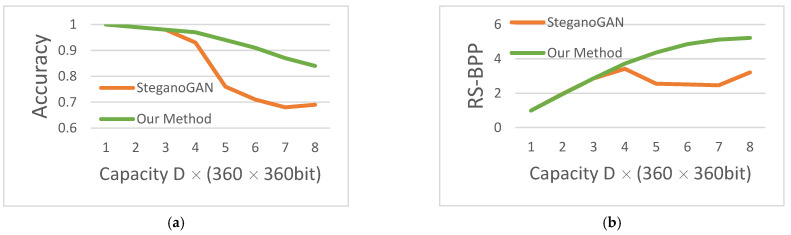
The accuracy and RS-BPP. (**a**) Decoding accuracy; (**b**) Reed-Solomon bits per pixel.

The accuracy and RS-BPP comparison dashboards of our method with SteganoGAN are shown in Figure 10, and the data are obtained from Table 1. The orange dash is the experimental data reproduced from the public code of SteganoGAN, and the green dash is the experimental data of our method. In Figure 10, it can be seen that the green line representing our method always leads, and Accuracy and RS-BPP lead the most in the high capacity case of hidden capacity.

**Figure 11:**
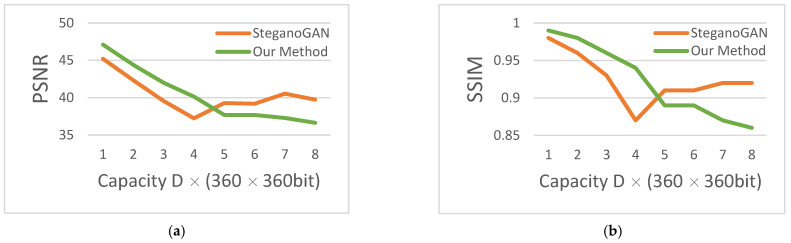
PSNR and SSIM. (**a**) Peak signal to noise ratio; (**b**) structural similarity Index.

In terms of image quality, the PSNR and SSIM comparison of our method with SteganoGAN is shown in Figure 11, and the data are obtained from Table 1. Because of the balance of transparency and capacity, the transparency should have decreased with the increase of capacity. However, SteganoGAN exceeds the limit performance, and thus the PSNR curve starts to rise. The PSNR curve and SSIM curve of our method were basically leading before this.

## 4. Conclusions

In this paper, AFHS-GAN is proposed. Firstly, it is pointed out that the current steganography methods based on deep learning have the problems of low hidden capacity and poor image quality. Secondly, the defects of simple deep network structure and underutilization of frequency-domain information in mainstream methods are analyzed. Then, AFcaNet and AFHS-GAN are presented in detail, including the design of the network structure and low-frequency loss function. Finally, the comparison results with the SteganoGAN method under various metrics are shown and analyzed. Experimental results show that our method improves the highest payload by 1.8 bits per pixel compared to SteganoGAN, and also improves the image quality on the Div2k dataset.

## Figures and Tables

**Table 1:** A comparison of our method and SteganoGAN with different capacities.

*D*	Accuracy		RS-BPP		PSNR		SSIM	
	Stegano GAN	Our Method	Stegano GAN	Our Method	Stegano GAN	Our Method	Stegano GAN	Our Method
1	1.00	1.00	0.99	0.99	45.20	47.10	0.98	0.99
2	0.99	0.99	1.96	1.94	42.33	44.39	0.96	0.98
3	0.98	0.98	2.86	2.86	39.56	42.00	0.93	0.96
4	0.93	0.97	3.42	3.72	37.23	40.14	0.87	0.94
5	0.76	0.94	2.55	4.37	39.28	37.68	0.91	0.89
6	0.71	0.91	2.51	4.86	39.19	37.69	0.91	0.89
7	0.68	0.87	2.46	5.12	40.54	37.29	0.92	0.87
8	0.69	0.84	3.21	5.22	39.73	36.64	0.92	0.86
